# Effects of scoparone on non-alcoholic fatty liver disease revealed by RNA sequencing

**DOI:** 10.3389/fendo.2022.1004284

**Published:** 2022-09-09

**Authors:** Xiaoyan Huang, Ya Gao, Houkang Cao, Jun Li, Siyi Mo, Ting Li, Jianzhao Wu, Kai Guo, Riming Wei, Kefeng Zhang

**Affiliations:** ^1^ Pharmacology Laboratory of Prevention and Treatment of High Incidence of Disease, Guilin Medical University, Guilin, China; ^2^ Guangxi Key Laboratory of Diabetic Systems Medicine, The Second Affiliated Hospital of Guilin Medical University, Guilin, China; ^3^ Department of Obstetrics & Gynaecology, National University of Singapore, Singapore, Singapore

**Keywords:** scoparone, non-alcoholic liver disease (NAFLD), ribonucleic Acid (RNA) sequencing, fatty acid (triglyceride) metabolism, cholesterol metabolism

## Abstract

Scoparone (SCO) is known to have curative effect of alleviating liver injury. The purpose of this study was to observe the therapeutic effect and possible mechanism of SCO against high-fat diet (HFD) induced non-alcoholic liver disease (NAFLD) through *in vivo* experiments and RNA sequencing. Male Kunming mice were fed with HFD for 8 weeks to establish a mouse model of NAFLD, and SCO was used to treat NAFLD. Histopathology and biochemical indicators were used to evaluate the liver injury and the efficacy of SCO. RNA sequencing analysis was performed to elucidate the hepatoprotective mechanism of SCO. Finally, the differentially expressed genes of cholesterol synthesis and fatty acid (triglyceride) synthesis pathways were verified by quantitative real-time polymerase chain reaction (qRT-PCR) and western blot. The histopathological results showed that HFD could lead to significant steatosis in mice, while SCO could alleviate liver steatosis remarkably in NAFLD mice. The determination of biochemical indicators showed that SCO could inhibit the increased serum transaminase activity and liver lipid level induced by HFD. RNA sequencing analysis of liver tissues found that 2742 and 3663 genes were significantly changed by HFD and SCO, respectively. SCO reversed the most of genes involved in cholesterol synthesis and fatty acid (triglyceride) metabolism induced by HFD. the results of the validation experiment were mostly consistent with the RNA sequencing. SCO alleviated liver injury and steatosis in NAFLD mice, which may be closely related to the regulation of cholesterol and fatty acid (triglyceride) metabolism.

## Introduction

Non-alcoholic fatty liver disease (NAFLD) is a chronic liver injury strongly linked to insulin resistance and genetic susceptibility. There is overwhelming evidence that NAFLD often accompanied by risks of obesity, dyslipidemia, and diabetes (1). The main pathological features of NAFLD were hepatocyte steatosis and excessive lipid accumulation in the liver (2). Severe NAFLD can develop into non-alcoholic steatohepatitis (NASH), which would in turn lead to liver cirrhosis and hepatic carcinoma (3). Epidemiological studies of NAFLD show that the prevalence of NAFLD has steadily increased year by year in the past decade, and has now reached about 25% (4). Although significant progress has been made in the treatment of NAFLD, its specific nosogenesis has not been elucidated brightly. Therefore, it is extremely important to investigate its pathogenesis and find effective drugs for treatment.

Scoparone (SCO) also known as 6,7-dimethoxycoumarin, has antioxidant, anti-inflammatory and anti-tumor functions. More important, it helps relieve the hepatic glucose and lipid metabolism disorders, and reduces lipid deposition in hepatocytes (5). Some studies have found that SCO can effectively alleviate carbon tetrachloride-induced liver injury associated with hyperlipidemia (reduces total cholesterol (TC), triglyceride (TG), low-density lipoprotein cholesterol (LDL-C) levels in serum) (6). Other studies suggested that SCO also mitigate hepatic steatosis and fibrosis in NAFLD mice induced by choline deficiency diet (7), which means SCO maybe improve NAFLD by regulating lipid metabolism. Furthermore, our preliminary study also confirmed that SCO could down-regulate the expressions of alanine aminotransferase (ALT), aspartate aminotransferase (AST), γ-glutamyl transferase (γ-GT) in serum, effectively improving liver damage (8). Up till now, although some drugs (such as vitamin E, statins and metformin) have been tried to treat NAFLD, the efficacy is limited (9–11). In other words, there are no clinically approved drugs for the treatment of NAFLD. Hence, we hypothesized that SCO might help alleviate HFD-induced NAFLD.

More and more evidence also indicated that the pathogenesis of NAFLD is a very complex and continuous process, and the specific pathological mechanism has not been fully elucidated (12). Although SCO has the effects of hepatoprotection and lipid-lowering, the underlying molecular mechanisms of mitigating NAFLD still remain unknown. As a high-throughput sequencing technology, RNA sequencing can more systematically reveal the pathogenesis of disease. As a consequence, RNA sequencing is widely used in basic research, clinical diagnosis, and drug development or other fields (13). Besides, RNA sequencing has been widely used in liver disease research, involving the screening of tumor markers or therapeutic targets and the elucidation of drug action mechanisms (14). By reason of the foregoing, RNA sequencing was applied to transcriptome analysis of mouse liver in this study to further clarify the molecular regulation mechanism of SCO, which can provide a basis for the application of SCO in clinical treatment of NAFLD.

## Materials and methods

### Ethical approval

All experiments in this study were performed in accordance with the guidelines of the Animal Experimentation Ethics Committee of the Guilin Medical University (approval No. 2020-0053).

### Drug preparation

SCO was purchased from Shanghai yuanye Bio-Technology Co., Ltd (HPLC ≥ 98%, Shanghai, China). Silymarin was provided by Dalian Meilun Biotechnology Co., Ltd. (UV ≥ 80%, Dalian, China).

### HFD-induced NAFLD and SCO administration

Male Kunming mice, 6-8 weeks old, weighing 18-20 g, were purchased from Hunan SJA Laboratory Animal Co., Ltd. All mice were given standard diet and water under the conditions of constant temperature (22 ± 2°C) and relative humidity (45%-50%). The mice were adaptively fed for 1 week before the experiments.

**Figure 1:**
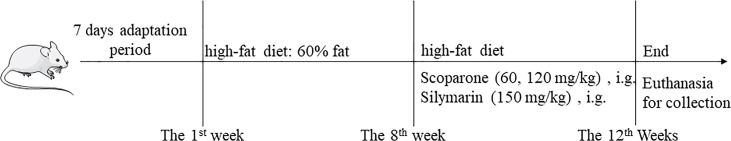
Overview of the NAFLD model procedure.

Firstly, 63 Kunming mice were randomly divided into 3 groups: Control group (n = 10), SCO group (n = 10) and model group (n = 43). The mice in the Control group and SCO group were given normal diet (provided 13.8% of calories from fat, 63.4% of calories from carbohydrates, and 22.8% of calories from protein (Jiangsu Xietong Pharmaceutical Bio-engineering Co., Ltd., Jiangsu, China)). The SCO group was administered intragastrically with SCO (120 mg/kg) for the next 12 weeks in order to determinate the toxic effect of SCO (Physiological saline was used as the solvent of SCO, and the mice in the Control group were administered intragastrically with the same volume of normal saline.). The model group was given a HFD (provided 60% of calories from fat, 20% of calories from carbohydrates, and 20% of calories from protein (Jiangsu Xietong Pharmaceutical Bio-engineering Co.,Ltd., Jiangsu, China)). After 8 weeks, 3 mice were randomly selected from the model group for pathological examination to evaluate whether the NAFLD model was successfully established (15). After confirming the successful establishment of NAFLD model, the model group was randomly divided into HFD group, HFD + SCO 60 group, HFD + SCO 120 group and HFD + Silymarin group (as a positive control), respectively, with 10 mice in each group. The HFD + SCO 60 group, HFD + SCO 120 group and HFD + Silymarin group were administered intragastrically with SCO (60 mg/kg, 120 mg/kg) and Silymarin (150 mg/kg) for 4 weeks (**Figure 1**), respectively. The dose of SCO was according to other studies and our previous studies with some adjustments (7, 8). At the end of the 12th week, all mice were fasted overnight. After 16 h, the mice were anesthetized for collecting blood and tissue samples. Some liver tissues and other tissues (brain, heart, lung, spleen and kidney) were collected and fixed in 4% paraformaldehyde (Beijing Solarbio Science & Technology Co.,Ltd., Beijing, China). In addition, some liver tissues used for Oil Red O staining, qRT-PCR and western blot were stored in -80°C.

### Hematoxylin-eosin (HE) and Oil Red O staining of tissues

Fixed for 48 h, liver, brain, heart, lung, spleen and kidney tissues were dehydrated and embedded in paraffin. Then, the tissues were cut into sections (4 μm) by microtome (Thermo Fisher Microm, Germany) and stained with HE. Subsequently, sections were treated with xylene and different concentrations of ethanol, and sealed with neutral resin after being cleared with xylene.

Meanwhile, the freezed liver tissue was sectioned (4 μm) by a freezing microtome (Jinhua Yidi Medical Equipment Co.,Ltd., China). Oil Red O staining was performed, and the slides were fixed with glycerol. Finally, the pathological structure and lipid deposition of mice tissues were observed and photographed with an optical microscope BX51 Optical microscope (Olympus, Japan).

### Indexes determination of serum and liver tissue samples

The expressions of liver function indexes (ALT, AST, γ-GT) and liver lipid indexes [TG, TC, high-density lipoprotein cholesterol (HDL-C) and LDL-C)] in serum were determined according to the kit instructions (Nanjing Jiancheng Bioengineering Institute Co.,Ltd., Nanjing, China). The OD value was detected by Epoch2 microplate reader (Biotek, USA).

### RNA sequencing analysis

Total RNA from liver tissues (Control group, HFD group and HFD + SCO group, 3 samples in each group) was extracted according to the instructions of Trizol kit (Life Technologies, California, USA). Briefly, mRNA was purified from total RNA by poly-T oligo-attached magnetic beads. Fragmentation was carried out using divalent cations under elevated temperature in First Strand Synthesis Reaction Buffer (5X). First strand cDNA was synthesized using random hexamer primer and M-MuLV Reverse Transcriptase, then RNaseH was used to degrade the RNA. Second strand cDNA synthesis was subsequently performed using DNA Polymerase I and dNTP. Remaining overhangs were converted into blunt ends *via* exonuclease/polymerase activities. After adenylation of 3’ ends of DNA fragments, Adaptor with hairpin loop structure were ligated to prepare for hybridization. In order to select cDNA fragments of preferentially 370~420 bp in length, the library fragments were purified with AMPure XP system (Beckman Coulter, Beverly, USA). Then PCR amplification, the PCR product was purified by AMPure XP beads, and the library was finally obtained. In order to ensure the quality of the library, the library needs to be tested. After the construction of the library, the library was initially quantified by Qubit 2.0 Fluorometer, then diluted to 1.5 ng/μl, and the insert size of the library is detected by Agilent 2100 bioanalyzer. After insert size meets the expectation, qRT-PCR was used to accurately quantify the effective concentration of the library (the effective concentration of the library is higher than that of 2 nM) to ensure the quality of the library.

Reference genome and gene model annotation files were downloaded from genome website directly. Index of the reference genome was built using Hisat2 (v2.0.5) and paired-end clean reads were aligned to the reference genome using Hisat2 (v2.0.5). feature Counts (v1.5.0-p3) was used to count the reads numbers mapped to each gene. And then FPKM of each gene was calculated based on the length of the gene and reads count mapped to this gene. The resulting P-values were adjusted using the Benjamini and Hochberg’s approach for controlling the false discovery rate. P adj ≤ 0.05 and Foldchange ≥ 1.5 were set as the threshold for significantly differential expressions.

### Validation experiments

#### Quantitative real-time polymerase chain reaction (qRT-PCR)

Liver tissues were lysed with trizol reagent (Beyotime Biotechnology, Shanghai, China) to obtain total RNA. After quantification of RNA by using NanoDrop 2000 (Thermo Fisher, Waltham, MA, USA), total RNA was reverse transcribed into cDNA according to the instructions of HiFiScript gDNA Removal cDNA synthesis Kit (Beijing ComWin Biotech Co.,Ltd., Beijing, China). Then, amplification was performed on a Quant Studio3 PCR machine (Thermo Fisher scientific, USA) according to the reaction conditions of the SYBR Green kit (Beijing ComWin Biotech Co.,Ltd., Beijing, China). The process of PCR reaction was at 95°C for 10 min, followed by 40 cycles (95°C for 15 s and 60°C for 1 min). 2−ΔΔCt was calculated according to the Ct value, and the relative expression was calculated by normalizing the expression of each group with GAPDH as a reference to normalize the expression of each group to calculate the relative expression (16). The sequences of primers are shown in **Table 1**.

**Table 1:** The primer sequences of mouse liver tissue.

Gene Name	Primer forward	Primer reverse
GAPDH	AGGTCGGTGTGAACGGATTTG	TGTAGACCATGTAGTTGAGGTCA
Prkaa1	GTCAAAGCCGACCCAATGATA	CGTACACGCAAATAATAGGGGTT
Prkaa2	CAGGCCATAAAGTGGCAGTTA	AAAAGTCTGTCGGAGTGCTGA
Srebf1	TGACCCGGCTATTCCGTGA	CTGGGCTGAGCAATACAGTTC
Hmgcr	AGCTTGCCCGAATTGTATGTG	TCTGTTGTGAACCATGTGACTTC
Fasn	GGAGGTGGTGATAGCCGGTAT	TGGGTAATCCATAGAGCCCAG
Acacα	GATGAACCATCTCCGTTGGC	GACCCAATTATGAATCGGGAGTG
Cpt1a	CTCCGCCTGAGCCATGAAG	CACCAGTGATGATGCCATTCT

#### Western blot

The liver tissues were lysed with RIPA that contained 1% phenyl methane sulfonyl fluoride (PMSF) (Beijing Solarbio Science & Technology Co.,Ltd., Beijing, China) for extraction of proteins. Bicinchoninic Acid Assay (BCA) kit (Beyotime Biotechnology, Shanghai, China) was used to detect the total protein concentration. Sodium dodecyl sulfate polyacrylamide gel electrophoresis (SDS-PAGE) sample loading buffer was added and protein denaturation was performed in a metal bath at 100°C for 10 min. Then, electrophoresis was used to separated protein samples. After that, proteins were transferred to polyvinylidene fluoride (PVDF) membranes (Millipore Corp, Billerica, MA, USA) at a constant current of 260 mA for 90 min in an ice bath. The PVDF membranes were then incubated with the following primary antibodies for 12 h at 4°C: AMP-activated protein kinase (AMPK) (1:1000, Abcam, UK), phosphorylation AMPK (p-AMPK) (1:5000, Abcam, UK), sterol regulatory element binding transcription factor 1 (SREBP-1c) (1:2500, Abcam, UK), carnitine palmitoyltransferase 1a (Cpt1a) (1:1000, Abcam, UK), acetyl-Coenzyme A carboxylase alpha (Acacα) (1:2000, Abcam, UK), fatty acid synthase (Fasn) (1:10000, Abcam, UK) and glyceraldehyde-3-phosphate dehydrogenase (GAPDH) (1:20000, Proteintech, UK). PVDF membranes were shaken overnight at 4°C, washed with TBST, and incubated with horseradish peroxidase-conjugated goat anti-mouse antibody or goat anti-rabbit antibody (Sam Golden Bridge biotechnology Co, Beijing, China) for 1.5 h at room temperature. Finally, the PVDF membranes were imaged in the Gene Gnome XRQ professional chemiluminescence imaging system (Syngene, USA), and the gray values of proteins were analyzed with Image-J 6.0 software. The target protein was divided by the gray value of the reference protein and normalized (17).

### Statistical analysis

IBM SPSS 20.0 software were used for statistical analysis, and data were expressed as mean ± standard (SD). Normal distribution was assessed by the Shapiro-Wilk normality test. For two-group comparisons with equal variance as determined by the F-test, an unpaired two-tailed t-test was used. Non-normally distributed data were analyzed by the Mann-Whitney U test. For comparisons between multiple groups, normally distributed and equal variance data were performed by one-way analysis of variance (ANOVA). Non-normally distributed or unequal variance data were performed by Kruskal-Wallis univariate analysis. *P* < 0.05 were considered statistically significant.

## Results

### SCO alleviated HFD induced NAFLD

**Figure 2:**
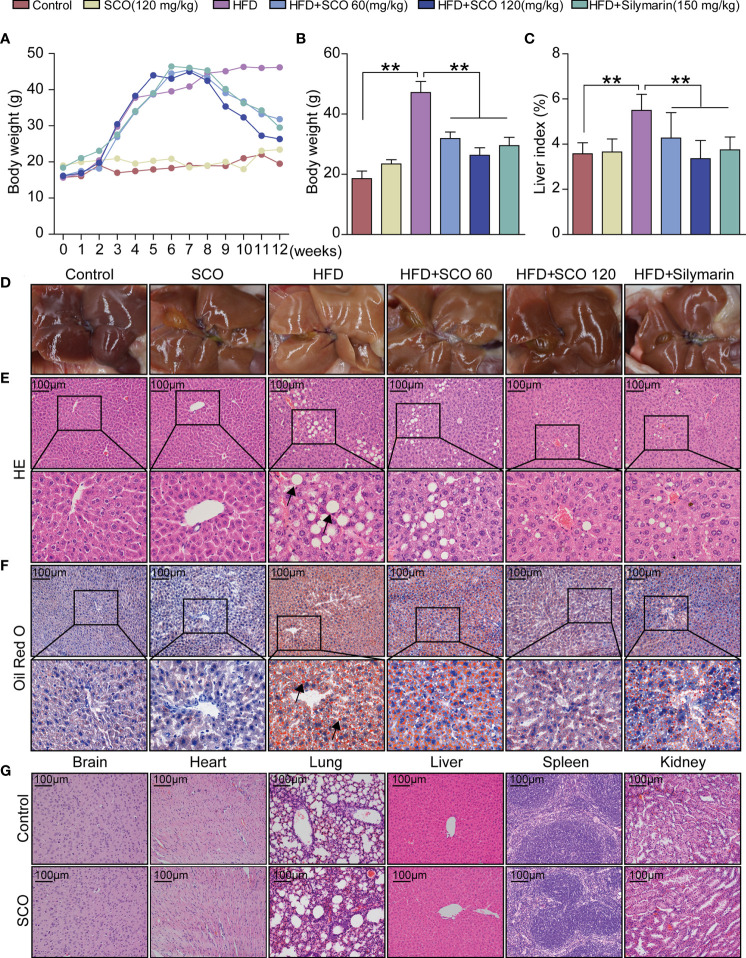
Pathological observation of main organs in mice. **(A–C)** Body weight and liver index of mice. **(D)** liver morphology. **(E, F)** Histological photomicrographs of liver sections stained with HE or Oil Red O (Scale bar = 100 μm), fat vacuoles and fatty degeneration (black arrow). **(G)** Histological photomicrographs of brain, heart lung, liver, spleen, kidney sections stained with HE (Scale bar = 100 μm). The HFD group was compared to the Control group; the HFD + SCO (60, 120 mg/kg) groups and HFD + Silymarin group were compared to the HFD group, ^**^ *P* < 0.01.

After 8 weeks of HFD modeling, the body weight of the mice increased significantly. Compared with the Control group, the body weight and liver index of the mice in the HFD group increased significantly at the twelfth week. After 4 weeks of SCO treatment, the body weight and liver index of the NAFLD mice were significantly decreased (**Figures 2A–C**). The liver of the Control group was bright red with a smooth surface, while the liver surface was pale yellow and the surface was greasy in the HFD group. As for the SCO 60 and SCO 120 groups, the liver color of the mice returned to bright and shiny. The results of HE and Oil Red O staining showed that HFD could cause obvious steatosis and lipid deposition in mouse liver tissue, while the number of fat vacuoles and lipid droplets were significantly reduced after 4 weeks of SCO administration (**Figures 2E, F**). At the same time, the HE staining of brain, heart, lung, liver, spleen and kidney tissues demonstrated that SCO had no significant toxic side effects on the liver and other tissues (**Figure 2G**).

### SCO inhibit lipid accumulation in NAFLD mice

**Figure 3:**
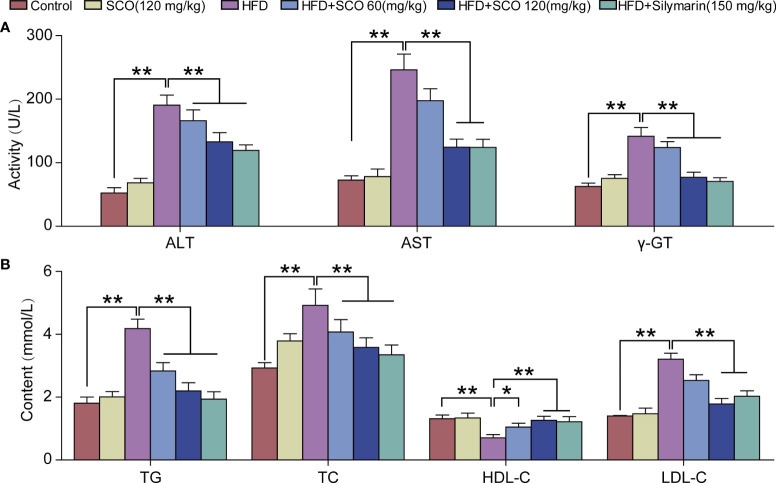
The results of detection of biochemical indicators. **(A)** Detection of serum liver function indicators of ALT, AST and γ-GT. **(B)** Detection of hepatic lipid indicators of TG, TC, HDL-C and LDL-C. All data are presented as the mean ± SD (n = 10). The HFD group was compared to the Control group; the HFD + SCO (60, 120 mg/kg) groups and HFD + Silymarin group were compared to the HFD group, ^*^ *P* < 0.05, ^**^ *P* < 0.01.

The measurement results of liver function indicators in serum showed that compared with the Control group, SCO (120 mg/kg) intervention had no significant effect on the indicators of liver function in serum in mice. After HFD inducing, the activities of ALT, AST, and γ-GT in serum were increased significantly, while the activities of ALT, AST, and γ-GT in serum were reduced markedly after SCO intervention (**Figure 3A**).

Briefly, SCO had no significant effects on serum lipid content in normal mice. HFD could significantly increase the content of TG, TC and LDL-C in serum, while the level of HDL-C was reduced. After SCO intervention, the content of TG, TC and LDL-C in serum were significantly decreased in NAFLD mice, while the content of HDL-C was increased (**Figure 3B**). To summarize, SCO alleviates NAFLD possibly by ameliorating liver injury and inhibiting lipid accumulation.

### The analysis of all differentially expressed genes

**Figure 4:**
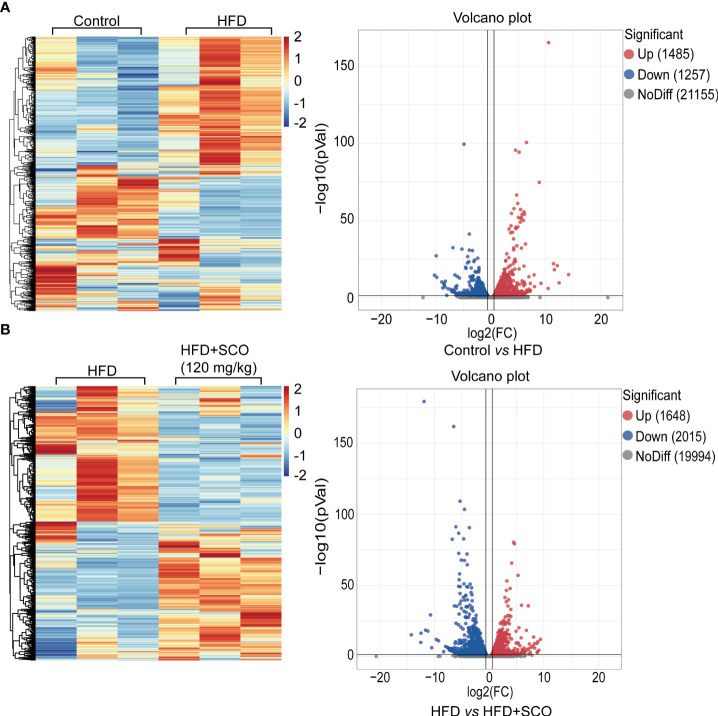
The hierarchical cluster analysis and scatter plot of all differentially expressed genes. **(A)** The Control group *vs* the HFD group. **(B)** The HFD group *vs* the HFD + SCO group.

The library quality evaluation results (detailed **Supplementary Information**) showed that the library constructed in this study was of high quality and reliability, which could be used for subsequent bioinformatics analysis. The results of differentially expressed genes analysis showed that compared with the Control group, a total of 2742 genes with significant changes in the HFD group, of which 1485 were up-regulated and 1257 were down-regulated (**Figure 4A**). Compared with the HFD group, 3663 genes were significantly changed after SCO intervention, of which 1648 genes were up-regulated and 2015 genes were down-regulated (**Figure 4B**).

### Effects of SCO on differentially expressed genes of cholesterol biosynthesis in liver tissue of NAFLD mice

**Figure 5:**
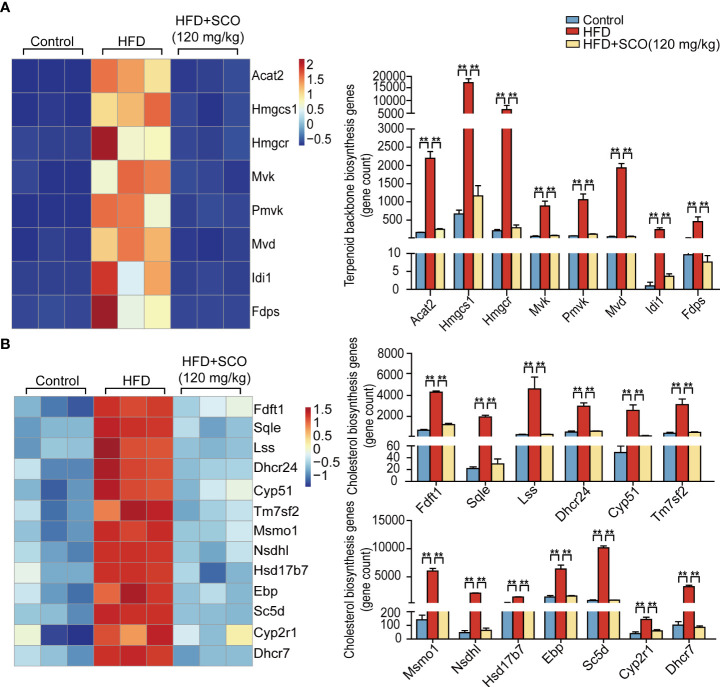
The differentially expressed genes of cholesterol biosynthesis in liver tissue. **(A)** Terpenoid backbone biosynthesis genes. **(B)** Cholesterol biosynthesis genes. ** Fold change ≥ 1.5 and *P* < 0.01.

Terpenoid backbone biosynthesis pathway (**Figure 5A**): the expressions of acetyl- Coenzyme A acetyltransferase 2 (Acat2), 3-hydroxy-3-methylglutaryl-Coenzyme A synthase 1 (Hmgcs1), 3-hydroxy-3-methylglutaryl-Coenzyme A reductase (Hmgcr), mevalonate kinase (Mvk), phosphomevalonate kinase (Pmvk), mevalonate (diphospho) decarboxylase (Mvd), isopentenyl-diphosphate delta isomerase (Idi1) and farnesyl diphosphate synthetase (Fdps) were significantly up-regulated in the HFD group, while these genes significantly down-regulated after SCO intervention.

Cholesterol biosynthesis pathway (**Figure 5B**): the expressions of farnesyl diphosphate farnesyl transferase 1 (Ftft1), squalene epoxidase (Sqle), lanosterol synthase (Lss), 24-dehydrocholesterol reductase (Dhcr24), cytochrome P450, family 51 (Cyp51), transmembrane 7 superfamily member 2 (Tm7sf2), methylsterol monoxygenase 1 (Mmo1), NAD(P) dependent steroid dehydrogenase-like (Nsdhl), hydroxysteroid (17-beta) dehydrogenase 7 (Hsd17b7), phenylalkylamine Ca2+ antagonist (emopamil) binding protein (Ebp), sterol-C5-desaturase (Sc5d), cytochrome P450, family 2, subfamily r, polypeptide 1 (Cyp2r1) and 7-dehydrocholesterol reductase (Dhcr7) were remarkably up-regulated in the HFD group, while the expressions of these genes were significantly down-regulated after SCO intervention.

### Effects of SCO on differentially expressed genes of fatty acid metabolism in liver tissue of NAFLD mice

**Figure 6:**
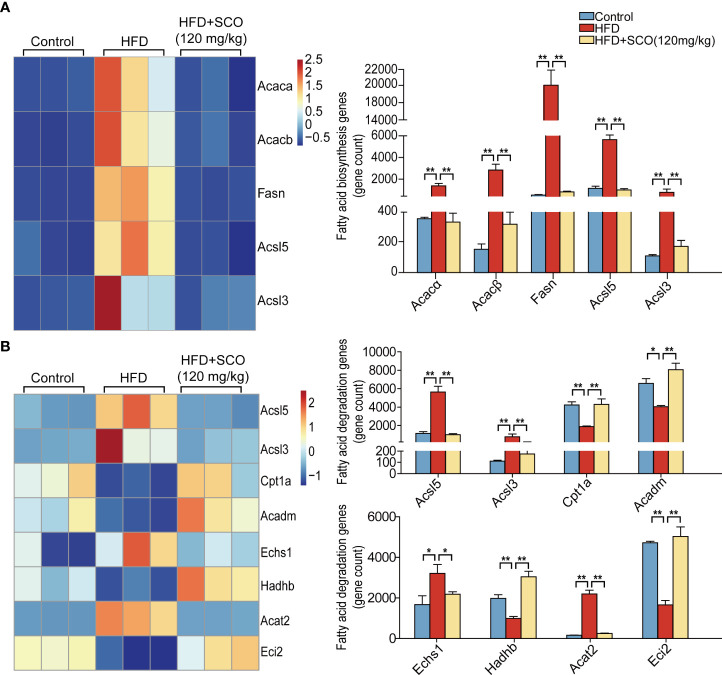
The differentially expressed genes of fatty acid metabolism in liver tissue. **(A)** Fatty acid synthesis genes. **(B)** Fatty acid (triglyceride) degradation genes. ^*^Fold change ≥ 1.5 and *P* < 0.05. ** Fold change = 1.5 and *P* < 0.01.

Fatty acid (triglyceride) synthesis pathway (**Figure 6A**): the expressions of Acacα, acetyl-Coenzyme A carboxylase beta (Acacβ), Fasn, CoA synthetase long-chain family member 5 (Acsl5) and acyl-CoA synthetase long-chain family member 3 (Acsl3) were significantly up-regulated in the HFD group, while SCO intervention suppressed the expressions of these genes.

Fatty acid (triglyceride) degradation pathway (**Figure 6B**): the expressions of Acsl5, Acsl3, enoyl Coenzyme A hydratase, short chain, 1 (Echs1) and Acat2 were significantly up-regulated in the HFD group, while the expressions of Cpt1a, acyl-Coenzyme A dehydrogenase, medium chain (Acadm), hydroxyacyl-Coenzyme A dehydrogenase/3-ketoacyl-Coenzyme A thiolase/enoyl-Coenzyme A hydratase (trifunctional protein), beta subunit (Hadhb) and coenzyme A delta isomerase 2 (Eci2) were down-regulated. After SCO intervention, the expressions of Acsl5, Acsl3, Echs1, and Acat2 were markedly down-regulated, while the expressions of Cpt1a, Acadm, Hadhb, and Eci2 were significantly up-regulated.

### Results of validation experiments

**Figure 7:**
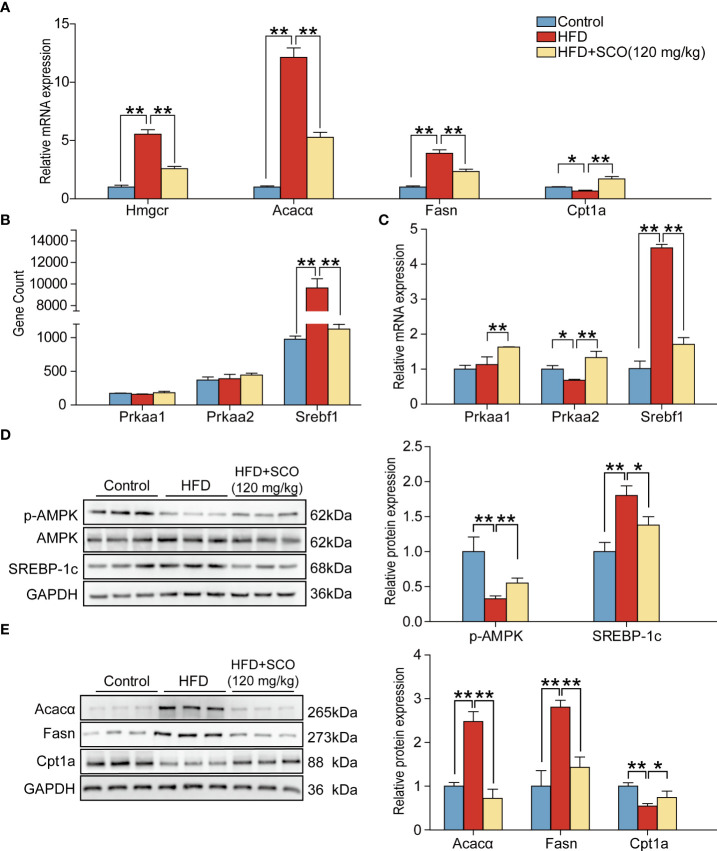
The results of validation experiments detected by qRT-PCR and western blot. **(A)** Relative mRNA expressions of Hmgcr, Fasn, Acacα and Cpt1a in mice liver tissue. **(B)** The gene expressions of Prkaa1, Prkaa2 and Srebf1. **(C)**: Relative mRNA expressions of Prkaa1, Prkaa2, Srebf1. **(D, E)**: The protein expressions of p-AMPK, SREBP-1c, Fasn, Acacα and Cpt1a. The relative expressions of protein were normalized against the control group, with GAPDH as the internal reference. All data are presented as the mean ± SD (n = 3). ^*^ *P* < 0.05, ^**^ *P* < 0.01.

To further verify the results of RNA sequencing, four representative genes were selected from the terpenoid backbone biosynthesis pathway, cholesterol synthesis pathway, fatty acid (triglyceride) synthesis pathway and fatty acid (triglyceride) degradation pathway, respectively. Then they were verified by qRT-PCR and western blot. The results of qRT-PCR experiments showed that the mRNA expressions of Acaca, Fasn and Hmgcr were significantly up-regulated in the HFD group, while the expression of Cpt1a was inhibited. SCO intervention significantly reversed the mRNA expressions of the above genes, respectively (**Figure 7A**). The results of western blot experiments indicated that the protein expressions of Acacα and Fasn in the HFD group were significantly up-regulated, while the expressions of Cpt1a were significantly down-regulated. After SCO intervention, the protein expressions of these genes were remarkably reversed, respectively (**Figure 7E**). All the above results were consistent with the RNA sequencing.

AMPK, encoded by Prkaa1 and Prkaa2, plays an important part in lipid metabolism, which can regulate the transcription factor SREBP-1c (encoded by Srebf1). SREBP-1c affects the downstream target genes (Acacα and Fasn). Therefore, we further detected the expressions of genes related to the AMPK pathway. RNA sequencing analysis suggested that the expression of Srebf1 was significantly up-regulated in the HFD group, while there were no significant changes of Prkaa1 and Prkaa2. The expression of Srebf1 was significantly down-regulated after SCO intervention. Interestingly, qRT-PCR and western blot results showed that SCO up-regulated the mRNA expressions of Prkaa1 and Prkaa2 and the protein expression of p-AMPK. And the expression of SREBP-1c was dramatically down-regulated in the HFD + SCO group (**Figures 7B–D**). These results suggested that SCO alleviate NAFLD might be related to regulating the expressions of AMPK and SREBP-1c.

## Discussion

Liver is the largest solid organ with the vital functions of catabolism and the metabolic balance of lipids (18). Dysfunction of hepatic lipid metabolism is an important cause of NAFLD. Excessive accumulation of lipid in the liver can induce hepatic steatosis, which further develops into NASH, giving rise to liver cirrhosis or hepatic carcinoma (19, 20). In our study, an *in vivo* NAFLD model was established induced by HFD, and the protective effects of SCO were explored by RNA sequencing and other methods. The results of HE and Oil Red O staining indicated that SCO significantly mitigated the hepatocyte steatosis and accumulation of lipid droplets. The results of RNA sequencing also suggested that HFD changed the expressions of many genes related to cholesterol synthesis and fatty acid (triglyceride) metabolism, while the expressions of these genes could be significantly reversed after SCO intervention.

When cell necrosis and other pathological changes occur in the liver, ALT, AST and γ-GT will overflow from the liver into the blood, resulting in abnormally elevated enzyme activity in plasma or serum (21). Therefore, the activities of ALT, AST and γ-GT can be used as indicators of liver function. In our study, activities of ALT, AST and γ-GT were significantly increased in the serum of HFD-induced mice, while SCO intervention could significantly reduce them (**Figure 3A**). Moreover, lipid metabolism is also closely related to NAFLD. As a lipid transporter, HDL is responsible for transporting cholesterol from outside the liver to inside the liver. And the increased expressions of LDL, TG, and TC have been proven to be closely related to the risk of NAFLD (22). In our study, the results of biochemical assay showed that SCO could significantly reduce the expressions of TG, TC and LDL-C, and increase the expression of HDL-C (**Figure 3B**). In short, the above results suggested that SCO has the function of improving liver injury. And its mechanism of alleviating HFD-induced NAFLD may be related to reducing lipid accumulation.

It is well known that obesity is one of the most distinctive features of NAFLD. Accumulation of a large amount of lipids in the liver can not only lead to disturbances in the systemic metabolic system, but also have deleterious effects on vital organs. Cholesterol and triglycerides are the main components of lipid accumulation. Hence, it is particularly important to study the metabolic process of cholesterol and triglyceride. Generally, the synthesis of cholesterol is mainly divided into two stages. The first stage is to synthesize the terpenoid backbone, which starts with the synthesis of terpenoids through the mevalonate pathway. During this stage, the expressions of differential genes related to terpenoid backbone synthesis (Acat2, Hmgcs1, Hmgcr, Pmvk, Mvd, and Fdps) was significantly up-regulated after HFD induction. The changes of these genes brought about increasing production of the intermediate product farnesyl pyrophosphate (23). However, the expressions of these genes were significantly down-regulated after SCO intervention (**Figure 5A**). In the second stage of cholesterol synthesis, HFD up-regulated the expressions of cholesterol synthesis related genes (Fdft1, Cyp51, Tm7sf2, Nsdhl, Msmo1, etc.), while the expressions of these genes were significantly down-regulated after SCO administration (**Figure 5B**). These results indicated that SCO may alleviate HFD-induced lipid accumulation by inhibiting the expressions of cholesterol synthesis-related genes.

In the *de novo* lipogenesis (DNL) pathway, new fatty acids (triglycerides) synthesize starts with acetyl-CoA in the liver. Acetyl-CoA converted to malonyl-CoA under the catalysis of ACC (Acacα and Acacβ), and then malonyl-CoA is converted to palmitate by Fasn catalyzing. Finally, the fatty acids are mainly stored as triglycerides (24, 25). In our study, HFD could significantly up-regulate the expressions of fatty acid (triglyceride) synthesis related genes (Acacα, Acacβ, and Fasn, etc.), while the expressions of these genes were inhibited after SCO intervention (**Figure 6A**). Mitochondrial fatty acid β-oxidation is the major pathway of fatty acid catabolism and plays a crucial role in maintaining energy homeostasis. After activation of Acsl5 and Acsl3, fatty acids will be synthesized into triglycerides or oxidatively degraded (26). Cpt1a is the enzyme that catalyzes a key rate-limiting step in mitochondrial fatty acid oxidation, and Cpt1a deficiency results in a reduced rate of fatty acid β-oxidation (27). Acadm, Echs1, Hadhb and Eci2 are important genes in the fatty acid β-oxidation pathway, which help to promote the degradation of fatty acids (triglyceride). The results of RNA sequencing indicated that the expressions of Acsl5, Acsl3, Echs1 and Acat2 were up-regulated in mice induced by HFD, while the expressions of these genes were down-regulated after SCO intervention. Besides, the expressions of Cpt1a, Acadm, Hadhb and Eci2 were up-regulated after SCO administration (**Figure 6B**), suggesting that SCO inhibit the progression of NAFLD may be closely related to regulating the process of fatty acid (triglyceride) synthesis and degradation.

AMPK plays an important regulatory role in lipid metabolism. It includes two protein subunits, namely AMPKα1 and AMPKα2, which are encoded by Prkaa1 and Prkaa2, respectively. AMPK can directly or indirectly regulate the expressions of lipid metabolism-related enzymes (Hmgcr, Fasn and Acacα, etc.) (28, 29). More importantly, its phosphorylation can promote lipid degradation and inhibit lipid synthesis. For example, AMPK can inhibit fatty acid synthesis by directly phosphorylating Acacα and Acacβ. Meanwhile, AMPK can also promote fatty acid oxidation by indirectly regulating Cpt1 activity (30). The transcription factor SREBP-1c plays an important role in lipid synthesis as well as an important downstream target gene of AMPK (31). Research has shown that SREBP-1c and SREBP cleavage-activating protein (SCAP) complex induce cholesterol biosynthesis in the liver by upregulating the gene expressions of cholesterol synthases (Hmgcr, Cyp51 and Dhcr7) (32). Besides, SREBP-1c regulates the transcription of downstream lipid synthesis-related genes Acacα and Fasn (33). In our study, the results of qRT-PCR and western blot demonstrated that SCO intervention could significantly up-regulate the expression of Prkaa1 and Prkaa2. Furthermore, SCO dramatically down-regulated the mRNA and protein expressions of SREBP-1c, Fasn and Acacα and up-regulated the protein expressions of Cpt1a and the p-AMPK (**Figure 7**). Therefore, we speculate that SCO may alleviate steatosis in NAFLD mice by regulating AMPK and SREBP-1c pathways.

## Conclusion

**Figure 8:**
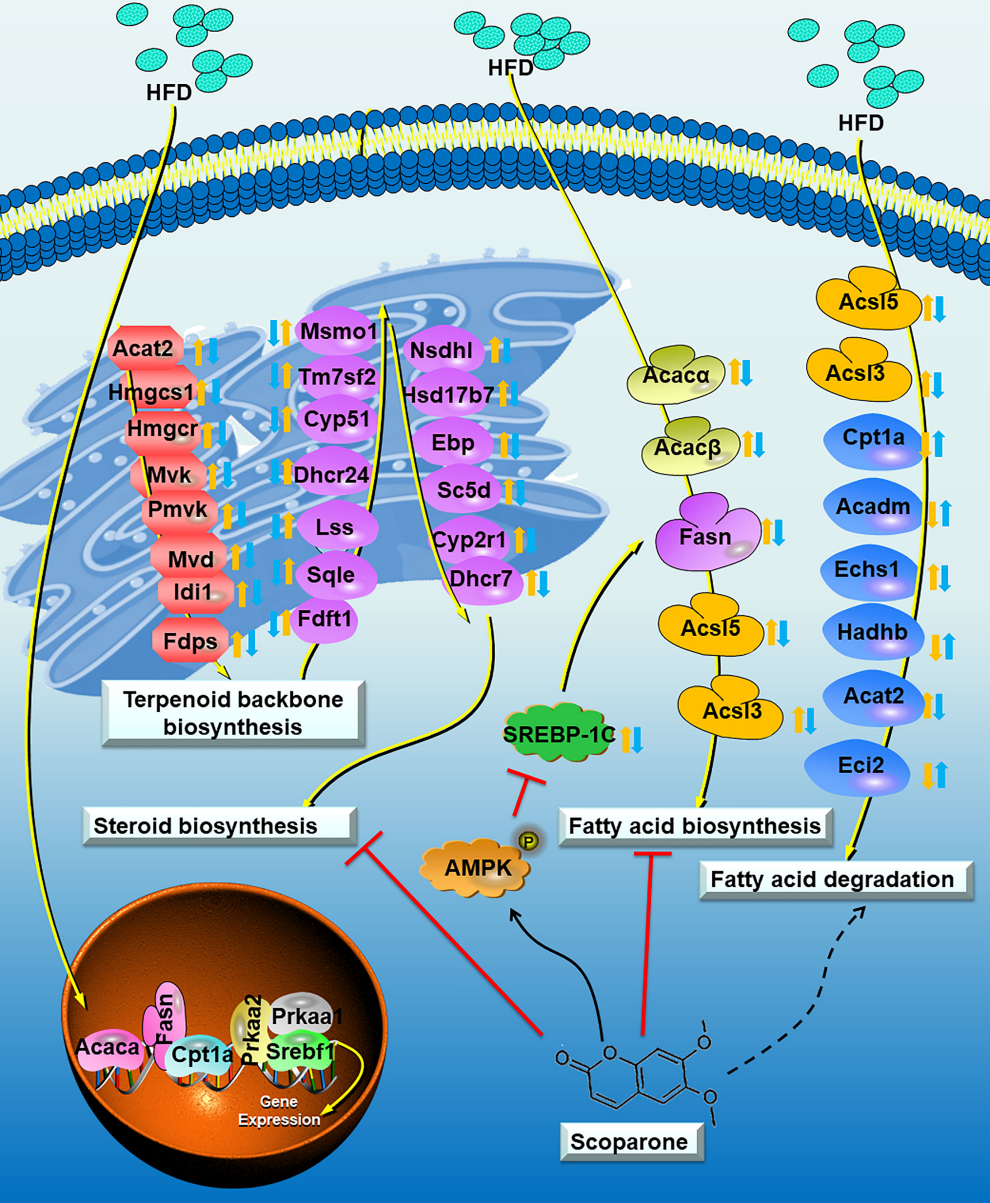
Proposed model depicting the process of cholesterol metabolism and fatty acid (triglyceride) metabolism in NAFLD mouse, and the underlying mechanisms of SCO in improving NAFLD. The Control group *vs* the HFD group: up-regulated:; down-regulated:. The HFD group *vs* the HFD + SCO group: up-regulated:; down-regulated:. Dashed arrows indicate that SCO may inhibit or promote the expression of these genes.

This study revealed the possible mechanism of SCO inhibiting NAFLD (**Figure 8**). In brief, SCO can effectively improve HFD-induced NAFLD in mice, and its mechanism may be interpersonally related to the regulation of cholesterol metabolism and fatty acid (triglyceride) metabolism in NAFLD mouse. The results of this study may provide a new strategy for clinical treatment of NAFLD.

## Data availability statement

The datasets presented in this study can be found in online repositories. The names of the repository/repositories and accession number(s) can be found in the article/**Supplementary Material**.

## Ethics statement

The animal study was reviewed and approved by the Animal Experimentation Ethics Committee of the Guilin Medical University

## Author contributions

RW and KZ: conceptualization and methodology. XH and YG: experimental studies and writing-original draft preparation. HC: statistical analysis. SM, TL, and KG: experimental studies and data acquisition. JL and JW: writing-reviewing, supervision, and editing.

## Funding

This study was supported by National Natural Science Foundation of China [No. 81960779, to YG, No. 82160811, to KZ].

## Acknowledgments

We thank everyone who contributed to this research, and apologize to colleagues whose work we could not cite due to space constraints.

## Conflict of interest

The authors declare that the research was conducted in the absence of any commercial or financial relationships that could be construed as a potential conflict of interest.

## Publisher’s note

All claims expressed in this article are solely those of the authors and do not necessarily represent those of their affiliated organizations, or those of the publisher, the editors and the reviewers. Any product that may be evaluated in this article, or claim that may be made by its manufacturer, is not guaranteed or endorsed by the publisher.
