# Supplementary material for: Effects of scoparone on non-alcoholic fatty liver disease revealed by RNA sequencing
Source: Front Endocrinol (Lausanne). 2022 Sep 9;13:1004284. doi: 10.3389/fendo.2022.1004284 (PMC9500212; doi:10.3389/fendo.2022.1004284)
Supplement: Supplementary file 1 [file Table_1.docx]

Supplementary Material

# Tables

**1.1 Library quality evaluation**

The sequencing data showed that the proportion of N-containing bases in all samples was 0.00%. The proportion of Q20 was higher than 98.00% and the proportion of Q30 was higher than 94.00% **(Table. S1)**, indicating that the quality of sequencing bases was high. The percentage (coverage) of Mapped Reads to Clean Reads in all samples reached over 93.00% **(Table. S2)** indicating that the reference genome assembly can meet the requirements of information analysis.

**Table. S1.** Sample sequencing data quality summary

| Sample ID | Raw_reads | Raw_bases | Clean_reads | Clean_bases | N (%) | Q20(%) | Q30(%) | GC_pct |
| --- | --- | --- | --- | --- | --- | --- | --- | --- |
| Control-1 | 44654894 | 6.7G | 41840448(93.70%) | 6.28G | 0.00 | 98.3 | 95.04 | 46.97 |
| Control-2 | 48504744 | 7.28G | 46184234(95.22%) | 6.93G | 0.00 | 98.27 | 94.96 | 45.71 |
| Control-3 | 47906490 | 7.19G | 44118328(92.09%) | 6.62G | 0.00 | 98.24 | 94.86 | 45.36 |
| HFD-1 | 47616622 | 7.14G | 45710596(96.00%) | 6.86G | 0.00 | 98.36 | 95.2 | 45.89 |
| HFD-2 | 45563100 | 6.83G | 43454378(95.37%) | 6.52G | 0.00 | 98.25 | 95.01 | 48.56 |
| HFD-3 | 46129460 | 6.92G | 44194798(95.81%) | 6.63G | 0.00 | 98.32 | 95.17 | 48.21 |
| HFD+SCO-1 | 47177026 | 7.08G | 43521068(92.25%) | 6.53G | 0.00 | 98.34 | 95.11 | 45.84 |
| HFD+SCO-2 | 45227968 | 6.78G | 42649846(94.30%) | 6.4G | 0.00 | 98.27 | 94.99 | 47.02 |
| HFD+SCO-3 | 45443378 | 6.82G | 41993674(92.41%) | 6.3G | 0.00 | 98.34 | 95.16 | 45.81 |

Note: Raw_reads: the number of reads in the raw data; Raw_bases: The number of bases in raw data (raw base=raw reads*150 bp); Clean_reads: The number of reads after filtering the original data; Clean_bases: The number of bases after filtering the original data (clean base=clean reads*150 bp); Q20: The percentage of bases with Phred value greater than 20 in total bases; Q30: The percentage of bases with a Phred value greater than 30 in the total bases; GC_pct: The percentage of G and C in the clean reads of the four bases. Phred = -10log10(e).

**Table. S2**. Sequence comparison results of sample sequencing data and selected reference genome

| Sample ID | Total_reads | Total_map | Unique_map | Multi_map | Reads Map to '+' | Reads Map to '-' |  |
| --- | --- | --- | --- | --- | --- | --- | --- |
| Control-1 | 41840448 | 39366153(94.09%) | 34338925(82.07%) | 5027228(12.02%) | 17174736(41.05%) | 17164189(41.02%) |  |
| Control-2 | 46184234 | 43681527(94.58%) | 36004576(77.96%) | 7676951(16.62%) | 18005279(38.99%) | 17999297(38.97%) |  |
| Control-3 | 44118328 | 41627036(94.35%) | 33958495(76.97%) | 7668541(17.38%) | 16974786(38.48%) | 16983709(38.5%) |  |
| HFD-1 | 45710596 | 43845845(95.92%) | 36793057(80.49%) | 7052788(15.43%) | 18410382(40.28%) | 18382675(40.22%) |  |
| HFD-2 | 43454378 | 41308584(95.06%) | 36901772(84.92%) | 4406812(10.14%) | 18467992(42.5%) | 18433780(42.42%) |  |
| HFD-3 | 44194798 | 41284605(93.42%) | 36120402(81.73%) | 5164203(11.69%) | 18072110(40.89%) | 18048292(40.84%) |  |
| HFD+SCO-1 | 43521068 | 40868273(93.9%) | 33813024(77.69%) | 7055249(16.21%) | 16910017(38.85%) | 16903007(38.84%) |  |
| HFD+SCO-2 | 42649846 | 40271093(94.42%) | 34024403(79.78%) | 6246690(14.65%) | 17017938(39.9%) | 17006465(39.87%) |  |
| HFD+SCO-3 | 41993674 | 39747931(94.65%) | 33048221(78.7%) | 6699710(15.95%) | 16526868(39.36%) | 16521353(39.34%) |  |

Note: Total reads: the number of Clean Reads, calculated as single-ended; Total_map: the number and percentage of reads aligned to the genome; Uniq Mapped Reads: the number of reads compared to the unique position of the reference genome and the clean Percentage of Reads; Multiple Map Reads: the number of reads compared to multiple positions in the reference genome and the percentage in Clean Reads; Reads Map to '+': the number of reads compared to the positive chain of the reference genome and the percentage in Clean Reads; Reads Map to '-': the number of reads compared to the negative strand of the reference genome and the percentage of Clean Reads.
