# Supplementary material for: Effects of scoparone on non-alcoholic fatty liver disease revealed by RNA sequencing
Source: Front Endocrinol (Lausanne). 2022 Sep 9;13:1004284. doi: 10.3389/fendo.2022.1004284 (PMC9500212; doi:10.3389/fendo.2022.1004284)
Supplement: Supplementary file 2 [file Table_2.docx]

Supplementary Material

## Supplementary Figure


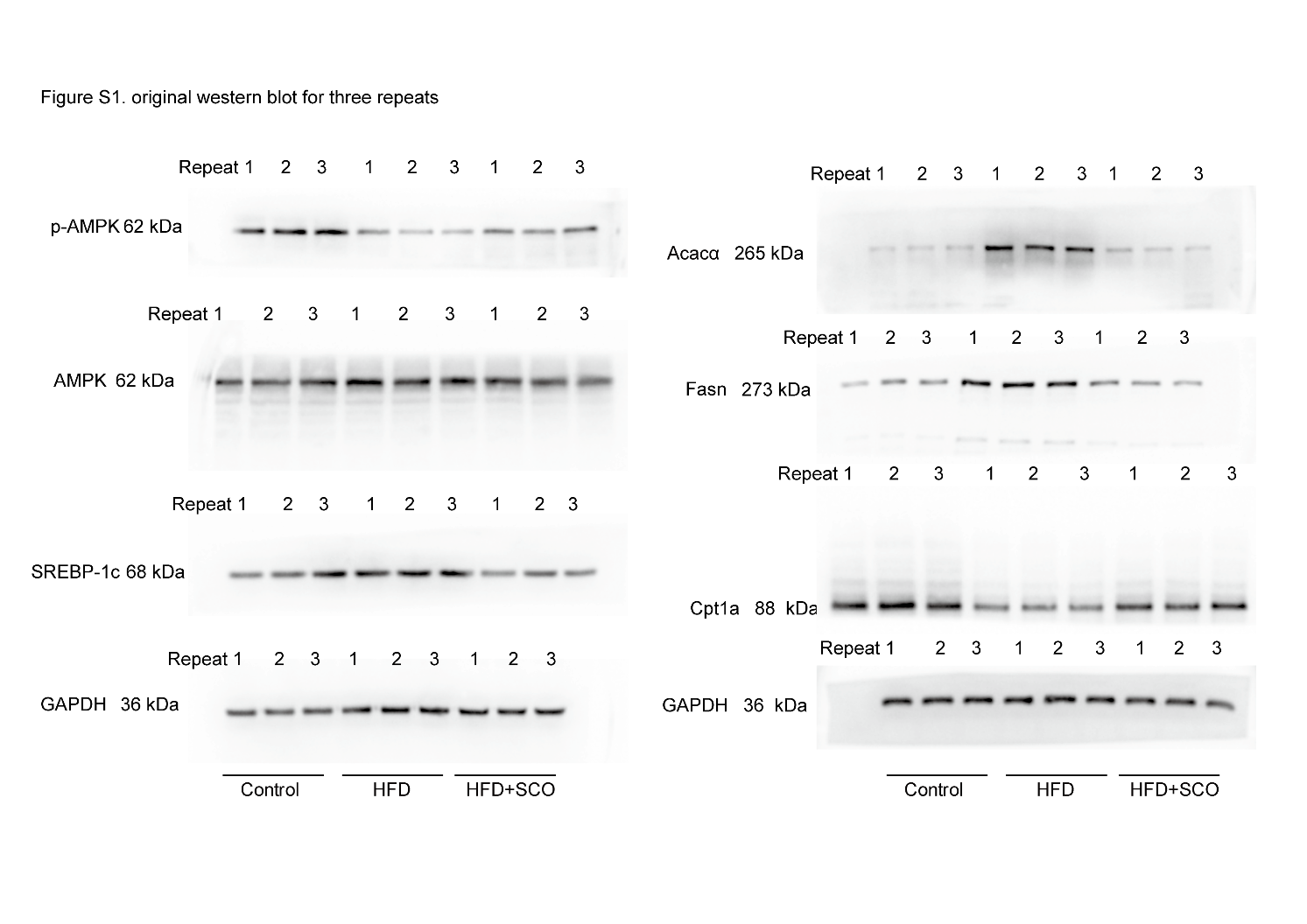


**Supplementary Figure 1.** original western blot for three repeats.
